# The impact of the coronavirus on African American unemployment: lessons from history

**DOI:** 10.1186/s12651-022-00308-5

**Published:** 2022-04-11

**Authors:** Ernst Coupet, Ehab Yamani

**Affiliations:** Department of Accounting and Finance, Chicago State University, 9501 S. King Drive, Chicago, IL 60628 USA

**Keywords:** E2, E3, J4, Labor markets, Unemployment, Financial crisis

## Abstract

In this article, our fundamental research question is to investigate the effect of the Coronavirus (named COVID-19) on the African American labor market. More specifically, we attempt to examine the potential economic impact of COVID-19 on the state of racial disparities among the African American labor market by examining two effects, namely, employment and income differentials, using national, state, and city level data (using data for all 77 neighborhood areas of the City of Chicago). Our central finding is that the labor market does not appear to treat black and white laborers as homogeneous, as attested by the finding that African American workers suffer from higher unemployment rates with higher volatility, lower median incomes, and they are more likely to work in the service sector, compared to their white counterparts, and we find this condition to be even larger in the City of Chicago. These findings have important policy implications.

## Introduction

The coronavirus is a rapidly evolving health pandemic that will have repercussions beyond individual health and the U.S. healthcare system. It has become clear that the outbreak of COVID-19 has disrupted the U.S. economy in general, and its economic impact on the labor market is unprecedented and highly uncertain making it more difficult for policymakers to formulate an appropriate policy response. Over decades, we find no other infectious disease outbreak that had more than a tiny effect on the U.S. labor market. It is notable that there will be a significant household and macroeconomic impact as this virus have generated large reductions in employment and earnings in the U.S. labor market and thus triggering an economic recession.

This, however, is only a partial effect on the labor market. While the virus shock will affect household employment and income, we anticipate that the economic impact is not likely to be equal across different racial groups among U.S. workers who will experience these disruptions differently. The existing literature emphasizes unemployment differentials (Hellerstein et al., 2008; Boustan and Margo, 2009; Bond and Lehmann, 2018; Yu and Sun, 2019; Button and Walker, 2020; Couch et al., 2020; Kim et al., 2021; Macartney et al., 2021; Mandel and Semyonov, 2021) as well as income differentials (Tangentially, Ileanu, and Tanasoiu, 2008; Raymond, 2018; Abdul Khalid and Yang, 2021; Chantreuil et al., 2021; Contreras et al., 2021; Ren, 2022) among different racial groups of workers.

In this article, we are motivated by such research on racial disparities and our goal is to examine the effect of the Coronavirus may have had on the state of racial disparities among the African American labor community. To better understand the possible racial disparities, we attempt to quantify the potential economic impact of COVID-19 on the African American labor market by controlling for two effects, namely, employment differentials and income differentials.

First, employment differentials effect. Under this analysis, we classify COVID-19 as an external shock (i.e., an unplanned and unexpected event) that can have a substantial impact on the labor market. Using monthly and quarterly U.S. unemployment data defined by race, we analyze the short-run effect of an exogenous shock by testing for Granger causality using cointegration and error-correction models. For comparative measures, we predict the unemployment differentials effect by drawing comparisons to the two most recent economic recessions: the terrorist attack on September 11, 2001 (9/11) and the 2008 global financial crisis. The rationale for examining these two historic recessions is to learn how different racial groups might be impacted by exogenous shocks in two different scenarios, and therefore, we can predict how different racial groups might fare from a recession that may follow the COVID-19 pandemic.^1^

Second, income differentials effect. We extend our analysis further and present a comparative income differential analysis across various racial groups in the City of Chicago. To this end, we use data for all 77 neighborhood areas of the City of Chicago, to better quantify the effect of the virus may have had on the black employment in the south and southeast sides of the city of Chicago, which are mostly populated by African Americans. We apply a traditional earnings function model to understand the net effect of the COVID-19 on the South and Southeast sides of the of City which are populated mostly by African Americans.

Our main finding is that firms in the labor market appear to prefer white employees to African American and Hispanics, suggesting that firms do not treat these laborers from the two markets as homogeneous. This conclusion is attested by several interesting findings that emerge from our unemployment differentials and income differentials analyses.

First, our unemployment differentials results show that the level of national African American unemployment is nearly twice that of the white unemployment over the entire full sample period. Furthermore, while the two examined recession episodes (i.e., the September 11 terrorist attack and the 2007–2008 recession) experienced exogenous shocks to the labor market and led to significant increases in the unemployment rates in all sectors, the increase in unemployment rate in the white sector paled to that of the African American sector.

Along the same lines, we also find that white unemployment Granger-causes African American unemployment, indicating a long-run association between white unemployment and African American unemployment, in the sense that unemployment is first decreased in the white sector, followed by a lagged unemployment decrease in the African American labor market. This finding suggest that most of the unemployment in the white sector are of the structural and frictional forms, while the African American unemployment is largely cyclical in nature. Put differently, the African American labor market appears to serve as a secondary labor market to the white sector that fills in during expansionary times but suffers great losses during economic downturns.

Second, our income differentials analysis results show that such observed racial disparities are even larger among the African American labor community in the City of Chicago. We find that African American workers in the Southeast and South sides of Chicago suffer from higher unemployment rates with higher volatility, lower median incomes, and they are more likely to work in the service sector, compared to their counterparts in other parts of the City.

Our findings have important policy implications. While it is uncertain to know for sure what will be the effect of this purely healthy-related exogenous shock to the economy, the effect of the COVID-19 virus is certain to be deep and broad for the African Americans who suffer from higher unemployment rates and lower median incomes. To alleviate this expected hardship, targeted public policy should be introduced so that we must allocate funding and resources to where they are most needed, and policy recommendations must be reflective of this reality. A uniform policy approach will not address the varied needs of groups and communities.

Hence, we propose two targeted policy recommendations. First, we recommend stimulating private fixed capital formation in African American communities by providing guaranteed heavily subsidized loans to those investing in African American communities. Our second recommendation is to enforce fair wages to ensure equitable wages across the labor markets. There is an abundance of evidence suggesting that the marginal product of labor is not compensated equitably across various sectors of the labor market.

The remainder of the paper is organized as follows. Section 2 reviews the literature. Section 3 outlines the econometric methodology which we employ. Our data are presented in Sect. 4. Sections 5 reports and discusses our empirical results. Section 6 concludes.

## Literature review

### Employment differentials

Our work builds on the recent research on racial employment differentials among different groups of workers defined by race. An incomplete list includes Hellerstein et al. (2008), Boustan and Margo (2009), Bond and Lehmann (2018), Yu and Sun (2019), Button and Walker (2020), Couch et al. (2020), Kim et al. (2021), Macartney et al. (2021), and Mandel and Semyonov (2021).

Differences in unemployment rates between African American and whites have been an ongoing discussion and research topic. Lynch and Hyclak (1984) analyze the disparities among various groups in the labor market, and they find that the level of the natural rate of unemployment has changed over time with a rising labor force participation among non-traditional groups in the labor market. Robinson (2010) explains differences in the levels of unemployment between Blacks and Whites from a cultural perspective, in the sense that employers engage in employment discrimination based on tastes derived from “infotainment” to bias their hiring practices and contribute to the wage gap between the two groups. Mouw (2000) uses a fixed effects model to explain the increase in unemployment gap between minority groups using the spatial mismatch hypothesis. This theory hypothesizes that both residential segregation and job decentralization adversely affect employment opportunities of minorities.

Realizing that the unemployment gap is only one facet of the overall inequities that occur between racial communities, researchers have incorporated many factors in attempt to explain overall inequities. Raymond (2018), for example, utilizes simple regression models to control for various factors and find that race remains the strongest predictor of persistent negative equity in the southeastern U.S. Further, Nkomo and Ariss (2014) show that the historical origins of white privilege explain persistence in the racial divide in organizations and the American labor market. Prior research has also focused on the lack of job opportunities in African American communities that contribute to increased levels of long-term unemployment in the African American. Kaplan (1999), for example, examines the number of job opportunities within very small neighborhoods, and finds that they do not vary much from neighborhood to neighborhood among white neighborhoods, but African American communities fall short of their white counterpart.

### Income differentials

Our research is related to another strand of the literature which examines income inequality among various racial groups (Tangentially, Ileanu, and Tanasoiu, 2008; Raymond, 2018; Abdul Khalid and Yang, 2021; Chantreuil et al., 2021; Contreras et al. 2021; Ren, 2022).

Broadly presented, there are two strands of literature that explain employment and income differentials between African Americans and other sectors of the labor market, namely the white sector. The first strand of literature takes a micro approach and postulates that a laborer’s potential earnings are a function of investments in human capital (Becker, 1958; Mincer, 1958; Chiswick, 2003; Ileanu and Tanasoiu, 2008; Aali-Bujari et al., 2019). This body of literature evolved from the seminal works of Becker (1962) and Mincer (1958) who contributed to the study of labor economics by developing what is now known as the earnings function. Further, Aali-Bujari et al. (2019) use Mincer’s (1958) earnings function to conclude that the level of education among Mexicans magnifies the increase in income levels and enlarges the human capital.

The second strand of the literature, very deep and broad in scope, takes a macro approach to analyze the income differentials between African Americans and other sectors of the labor market. Raymond (2018) finds that race is the strongest predictor of persistent negative equity in the southeast of the U.S., even after controlling for factors relating to the 2008 crisis. Mouw (2000) analyzes unemployment rates in Chicago and Detroit by targeting spatial employment opportunities and residential housing. Using panel data and a fixed-effect model, Mouw (2000) finds that decentralization of employment and the loss of manufacturing jobs resulted in spatial distribution of employment in the two cities.

Relatedly, Immergluck (1998) looks at proximity of job opportunities in urban areas to explain unemployment rates among urban dwellers, and he finds that race and educational attainment have the largest effects on unemployment rates. Further, Hoynes et al. (2012) find that the net effect of the 2007–2008 recession on unemployment was not homogeneous across the various sectors of the labor market. Specifically, African Americans and Hispanics suffered higher levels of unemployment during this crisis.

## Methodology

### Employment differentials analysis

*Quantifying the Impact of COVID-19 on Labor Market:* Our goal is to examine the economic impact of COVID-19 by drawing comparisons to the recent recessions. We consider the impact of Coronavirus on the African American labor markets nationally (as well as in the state of Illinois) and compare it to the those during the two most recent economic recessions: the terrorist attack on the U.S. on September 11, 2001 and the global financial crisis in 2008.

*Labor Model:* We begin with a typical firm’s Cobb–Douglas production function with constant returns to scale of a firm at any given time can be expressed as:

1 $${Y}_{t}={{A}_{t}}^{\gamma }{{K}_{t}}^{\alpha }{{H}_{t}}^{\beta }{{L}_{t}}^{1-\gamma -\alpha -\beta }$$

where Y is each firm’s temporal output; A is the level of multifactor productivity; H is the level of human capital embodied and L is the level of employment. Each factor exhibits diminishing returns. That is: $\gamma ,\alpha ,and \beta are<1.$ Except for their racial makeup, workers are homogeneous. The firm’s labor force is diverse and consists of a vector of races and nationalities:

2 $${L}_{t}={{L}_{t}}^{AA}+{{L}_{t}}^{W}+{{L}_{t}}^{L}+{{L}_{t}}^{O}$$

where $AA$, $W$, $L$, and $O$ refer to the employment rates among African Americans, Whites, Latin, and others, respectively. To analyze the production function’s short-run dynamics, we take logs and differentiate Eq. (1) w.r.t. to time (for example, $\dot{Y}=\frac{dY}{dt})$. This yield:

3 $$\frac{\dot{Y}}{Y}=\gamma \frac{\dot{A}}{A}+\alpha \frac{\dot{K}}{K}+\left(1-\gamma -\alpha -\beta \right)\frac{\dot{L}}{L}+\beta \frac{\dot{H}}{H}.$$

Taking time derivatives of Eq. (2) and dividing by ${L}_{t}$ yields:

4 $$ \frac{{\dot{L}}}{L} = \frac{{\dot{L}^{{\dot{A}A}} }}{L} + \frac{{\dot{L}^{{\dot{W}}} }}{L} + \frac{{\dot{L}^{{\dot{L}}} }}{L} + \frac{{\dot{L}^{{\dot{O}}} }}{L} .$$

Substituting Eq. (4) into Eq. (3) yields Eq. (5):

5 $$ \frac{{\dot{Y}}}{Y} = \gamma \frac{{\dot{A}}}{A} + \alpha \frac{{\dot{K}}}{K} + \left( {1 - \gamma  - \alpha  - \beta } \right)\left( {\frac{{\dot{L}^{{\dot{A}A}} }}{L} + \frac{{\dot{L}^{{\dot{W}}} }}{L} + \frac{{\dot{L}^{{\dot{L}}} }}{L} + \frac{{\dot{L}^{{\dot{O}}} }}{L}} \right) + \beta \frac{{\dot{H}}}{H}. $$

Rearranging Eq. (5) for the employment growth of African American employment leaves:

6 $$\frac{{\ddot{L}^{AA}}}{L}=\frac{1}{\left(1-\gamma -\alpha -\beta \right)}\frac{\dot{Y}}{Y}-\frac{\gamma }{\left(1-\gamma -\alpha -\beta \right)}\frac{\dot{A}}{A}-\frac{\alpha }{\left(1-\gamma -\alpha -\beta \right)}\frac{\dot{K}}{K}-\frac{{{\ddot{L}}^{W}}}{L}-\frac{{\dot{L}}^{L}}{L}-\frac{{\dot{L}}^{o}}{L}-\frac{\beta }{\left(1-\gamma -\alpha -\beta \right)}\frac{\dot{H}}{H}$$

As Eq. (6) indicates, except for output growth, the coefficients of all the right-hand-side variables are negative. Holding all other factors constant, an increase in output causes an increase in the growth of African American employment. Because the level of employed labor is fixed any point in time, an increase in the employment rate of African Americans can only come from a reduction of employment in the other sectors, holding output constant. The purpose of the labor market study is two-fold. First, we analyze the differences in unemployment rates among three sectors of the labor market: African Americans, Whites, and Latin. In addition to differences in the levels of unemployment among the three sectors of the labor market, we will test for differential effects on unemployment rates resulting from exogenous shocks in the economy. To accomplish this, we will decompose the time into three periods around two monumental crises in contemporary American history. We will look at unemployment levels surrounding the September 11 attacks terrorist act and the Great Financial Recession. We will test for changes in the mean unemployment rates before and after exogenous shocks from the two crises.

*Unemployment Rate Levels Analysis:* Let ${{\overline{\mu }}_{t-j,t}}^{i}=$ average unemployment rate for the ith sector of the labor market from time t-j to t; ${{\overline{\mu }}_{t,t+k}}^{i}=$ average unemployment rate for the ith sector from the time of event, t, to time t + k, a later date; and ${\overline{\mu }}_{t-j,t}$ is defined as $logit u$, where $logit u=\mathrm{ln}(u/(1-u))$ given that unemployment rates are positive. If the fiscal and monetary stimuli work well to restore the labor market sector equilibrium from an exogenous shock, then ${{\overline{\mu }}_{t-j,t}}^{i}\ne {{\overline{\mu }}_{t,t+k}}^{i}$. For example, suppose the unemployment rate in a labor market is a%. As a result of an exogenous shock, the unemployment rate rises above a% to b%.

If the government and central bank prescribe the exact amount of intervention in the financial and capital markets, the average unemployment rate will be restored to a%. If workers are homogeneous, then the net effect on this sector should be the same for all other sectors of the labor market—that is, ${{\overline{\mu }}_{t-j,t}}^{i}-{{\overline{\mu }}_{t,t+k}}^{i}={{\overline{\mu }}_{t-j,t}}^{o}-{{\overline{\mu }}_{t,t+k}}^{o}$. If the market values one sector of the market over the other for any reason, then the differences in each unemployment level for the sectors will not converge. In this case, it may be that ${{\overline{\mu }}_{t-j,t}}^{i}-{{\overline{\mu }}_{t,t+k}}^{i}>{{\overline{\mu }}_{t-j,t}}^{o}-{{\overline{\mu }}_{t,t+k}}^{o}$.^2^

The dynamics of the labor market will be analyzed with a system of equations. Two non-stationary variables are cointegrated of order 1, CI (1,1), if their levels are nonstationary and stationary in their first difference. If so, we use the Johansen method to test for the rank of the system of equation to determine long-run relationships. If there is a long-run relationship, then we use a Vector Error-Correction Model (VECM) to establish the long-run and short-run causality between the variables. If the system is cointegrated, we use an error correction model of the form:

7 $$\Delta {\mu }_{i,t}={{\alpha +\beta }_{0}ec}_{i,t-1}+{\beta }_{1}\Delta {\mu }_{i,t-1}+{\beta }_{2}\Delta {\mu }_{j,t-1}+{e}_{i,t}$$

where ${\mu }_{i,t}$ is the unemployment rate at time t of one race, ${\mu }_{j,t}$ is the unemployment rate of another race at the same time, ${ec}_{i,t}$ is the error correction term from the previous period, and ${e}_{i,t}$ is the white noise error term in the current period. If the variables are not cointegrated, then we can establish a vector autoregression (VAR) model to test for short-run causality

8 $$\Delta {\mu }_{i,t}=\gamma +{\beta }_{3}\Delta {\mu }_{i,t-1}+{\beta }_{4}\Delta {\mu }_{j,t-1}+{e}_{i,t}$$

This will be followed by the impulse response function, establishing in the time domain the effect of an exogenous variable on the other variables.

### Income differentials analysis

*The Earnings Function*: In the second part of our analysis, we proceed with the development of the earnings function, followed by a labor market segment model. Mincer (1958) and Ileanu (2008) model the earnings function of an individual using the stylized general function as:

9 $$y=h\left(S,x,F\right)+\varepsilon $$

where $y$ is net earnings; $S$ is the years of schooling; and $x$ represents the years of experience; and $F$ is a vector of exogenous variables that are not related to investments in human capital, as defined in Eq. (1). A structural equation that is typically used to estimate earnings in Eq. (9) is:

10 $$y={S}^{\alpha }{H}^{\beta }{e}^{F}$$

where H refers to the number of years of experience and F is a vector of variables that are not related to human capital such as race, language, gender. Taking logs of Eq. (10), we get,

11 $$\mathit{ln}y=\alpha lnS+\beta lnH+F$$

Equation (11), known as the earnings function, is used to estimate an individual’s post investment earnings. We will estimate the coefficients of Eq. (11) for neighborhood area households in the City of Chicago with regression Eq. (12) below:

12 $$ln {y}_{i}={\widehat{\alpha }}_{0}+\widehat{\beta }ln{S}_{i}+{F}_{i}+{e}_{i}$$

*Essential Workers Sector:* The likelihood of working as an essential worker in the City of Chicago, denoted as $Ess$, is assumed to be a function of the educational level and other exogenous variables such as race, gender, and income, as follows

13 $$Prob(Ess)=f(Schooling,income,X)$$

where we assume the following relationships ex ante: ${{\partial \left( {Prob\left( {Ess} \right)} \right)} \mathord{\left/ {\vphantom {{\partial \left( {Prob\left( {Ess} \right)} \right)} {\partial Schooling}}} \right. \kern-\nulldelimiterspace} {\partial Schooling}} < 0;$ ${{\partial ^{2} \left( {Prob\left( {Ess} \right)} \right)} \mathord{\left/ {\vphantom {{\partial ^{2} \left( {Prob\left( {Ess} \right)} \right)} {\partial Schooling^{2} }}} \right. \kern-\nulldelimiterspace} {\partial Schooling^{2} }} > 0;$ ${{\partial \left( {Prob\left( {Ess} \right)} \right)} \mathord{\left/ {\vphantom {{\partial \left( {Prob\left( {Ess} \right)} \right)} {\partial Income}}} \right. \kern-\nulldelimiterspace} {\partial Income}} < 0. <0$. Essential service workers are deemed necessary functions for society. This includes emergency room healthcare providers in hospitals, customer service representatives in retail outlets, and emergency service providers such as firefighters, police, etc. We assume that the likelihood of working in the service sector decreases with the number of years of schooling. However, with increases in schooling beyond college, this likelihood increases. The nonlinearity incorporates emergency room healthcare providers. We also assume, a priori, the likelihood of being an essential service provider is a decreasing function of income—however, in an increasing rate.

## Data

We extract data from two databases: The Bureau of Labor statistics (BLS) and the Environmental Systems Research Institute (ESRI) databases. We use BLS to collect monthly data on the national unemployment rates (as well as quarterly data for the state of Illinois), while we use ESRI data to collect household level market-related information for all 77 neighborhood areas of the City of Chicago. Our entire annual sample period begins in January 1989 and covers the period until February 2020. Although we examine the unemployment rates over the full sample period spanning the period from January 1st, 1989 to February 1st, 2020, we focus our analysis on the periods before and after the terrorist attack on September 11, 2001 and the 2007–2008 global financial crisis, as the key events. For this, we examine two separate sub-periods around each crisis. These sub-periods are: (1) the pre-9/11 crisis period covers the period from January 1st, 1989 to September 11th, 2001; (2) the post-9/11 crisis period spans the period from September 11th, 2001 to February 1st, 2008; (3) the pre-2008 crisis period covers the period from January 1st, 2008 to November 1st, 2010; (4) the post-2008 crisis period spans the period from November 1st, 2010 to February 1st, 2020. Refer to Tables 1, 70, 120 and 130 for descriptive statistics of the data.

**Table 1:** Descriptive statistics on the monthly National U.S. Unemployment Rates

	African American	White	Latin	Total
Panel A. Full Sample Period—Jan/1/1989 to 2/1/2020				
N	374	374	374	374
Mean	10.61	5.11	7.92	5.81
Median	10.50	4.70	7.50	5.40
S.D	2.62	1.46	2.30	1.58
Max.	16.8	9.20	13.00	10.0
Min.	5.4	3.10	3.90	3.5
Panel B. 9/11 Subsample Period				
B.1. Pre-9/11 period—Jan/1/1989 to 9/11/2001				
N	143	143	143	143
Mean	10.81	4.85	8.66	5.58
Median	10.80	4.70	8.80	5.40
S.D	1.95	0.98	1.75	1.08
Max.	14.70	6.90	12.10	7.80
Min.	7.00	3.40	5.10	3.80
B.2. Post-9/11 Sample—9/11/2001 to 2/1/2008				
N	77	77	77	77
Mean	9.75	4.62	6.54	5.27
Median	9.80	4.60	6.60	5.40
S.D	0.97	0.48	0.98	0.55
Max.	11.50	5.50	8.30	6.30
Min.	7.60	3.80	4.80	4.40
Panel C. 2008 Global Financial Crisis Subsample Period				
C.1. Crisis Period—2/1/2008 to 11/1/2010				
N	35	35	35	35
Mean	13.56	7.44	10.67	8.20
Median	14.80	8.50	12.00	9.40
S.D	2.75	1.75	2.37	1.86
Max.	16.80	9.20	13.00	10.0
Min.	8.40	4.40	6.20	4.90
C.2.Post Crisis Period—11/1/2010 to 2/1/2020				
N	111	111	111	111
Mean	10.20	5.14	7.20	5.83
Median	9.40	4.50	6.60	5.20
S.D	3.36	1.65	2.53	1.85
Max.	16.5	8.50	12.90	9.30
Min.	5.40	3.10	3.90	3.50

## Empirical results

### Employment differentials results

#### Level shock analysis—the case of the United States

To set the stage, Table 1 provides the descriptive statistics of historical unemployment for the full sample and by race. From January 1989 to February 2020, the average monthly unemployment rate for African Americans is 10.61%, compared to 5.11% for the White Americans. This is more than twice the unemployment rate of White Americans and exceeds that of the Latino sector by approximately 34%. The standard deviation of 2.62% for the African American unemployment rate also significantly higher than that of the White American sector as well. This is an indication of the volatility of those unemployed. A higher level would be an indication that household employment levels are inconsistent, an indication that household income is volatile as well.

**Fig. 1:**
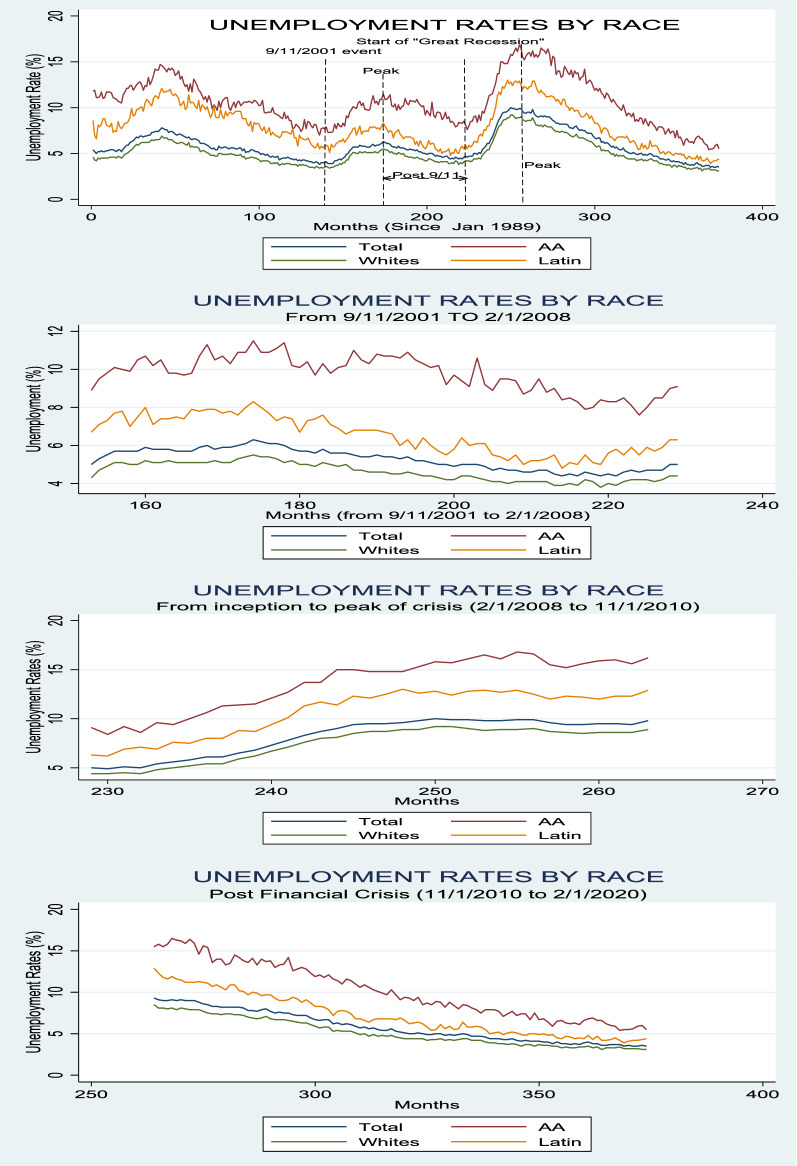
National unemployment rates by race

To get an understanding on the net effect of crisis on each sector of the labor market, Table 2 reports the mean differential for unemployment rates across various racial groups in the U.S. before and after each economic recession. Monthly African American unemployment rates for the 143 months prior to the 9/11 crisis was 13.56%, with a standard deviation of 2.75%. For the 77 months after the crisis, the average African American unemployment rate fell to 9.75%, a decrease of 1.06% which is statistically significant at the 1% level. In comparison, over the same months leading to the 9/11 crisis, White Americans averaged an unemployment rate of 4.85%. For the 77 months after the crisis, the unemployment rate fell to 4.62%, a 0.23% (1% p-value) decline. The 9/11 shock paled against the financial crisis of 2008. The exogenous shock of the financial crisis caused an increase of 3.81% in unemployment to a high of 13.56% in the African American sector. This is much higher than the effect on the White American sector which experienced a 2.82% increase in unemployment to a high of 7.44%. All the unemployment differential shocks are significant at the 1% level. It is notable that African Americans not only experience higher long-run equilibrium unemployment rates, but that exogenous shocks affect the African American labor market at a larger scale. To provide a visual illustration of Tables 1 and 2, Fig. 1 plots the time series fluctuations of national unemployment rates defined by race over the full sample period as well as the subsample periods.

**Table 2:** Mean differential analysis for unemployment rates in the U.S

Panel A. Pre-September 11—Post September 11 attacks means differential analysis				
A.1. African Americans				
	Pre-911 UER	Post-911 UER	Mean Differential	t-statistic (p-value)
Mean	10.81	9.75	-1.06	-5.38 (0.000)
S.D	1.95	0.97		
N	143	77		
A.2. White Americans				
	Pre-911 UER	Post-911 UER	Mean Differential	t-statistic (p-value)
Mean	4.85	4.62	− 0.23	− 2.33 (0.01)
S.D	0.98	.48		
N	143	77		

Panel B. Pre-2008- Post 2008 means differential analysis				
B.1 African Americans				
	Max 2008 UER	Post-2008 UER	Mean Differential	t-statistic (p-value)
Mean	13.56	10.2	− 3.36	− 5.96 (0.000)
S.D	2.75	3.36		
N	35	111		
B.2. White Americans				
	Max 2008 UER	Post-2008 UER	Mean Differential	t-statistic (p-value)
Mean	7.44	5.14	− 2.3	− 6.87 (0.000)
S.D	1.75	1.65		
N	35	111		

Panel C. 2018 crisis means differential analysis				
C.1. African Americans				
	Post 9/11 UER	Max 2018 Crisis UER	Mean Differential	t-statistic (p-value)
Mean	9.75	13.56	3.81	7.97 (0.000)
S.D	0.97	2.75		
N	77	35		
C.2. White Americans				
	Post 9/11 UER	Max 2018 Crisis UER	Mean Differential	t-statistic (p-value)
Mean	4.62	7.44	2.82	9.37 (0.000)
S.D	0.48	1.75		
N	77	35

### Market dynamics—the case of the United States

To analyze the dynamics of the labor markets, we examine whether markets are cointegrated. Cointegration requires that both series are non-stationary in their levels and stationary in their first difference. In Table 3, we run the Augmented Dickey-Fuller and Phillips-Perron Test with optimal lag length of 4 which was determined using the AIC (Information Criterion). Both tests show that we cannot reject the null hypothesis of a unit-root (non-stationarity) for the unemployment levels of the full sample. However, we reject the null hypothesis of unit root in their first difference at the 1% level. This criterion meets the minimum standard to test for cointegration among the two series. In Table 4, we report the results of the Johansen maximum likelihood test, and the Trace statistic suggests that the null hypothesis of no cointegration cannot be rejected at the 5% level for the full sample.^3^ Therefore, the two series are not cointegrated.

**Table 3:** Unit root tests for unemployment rates for the U.S

Variable	ADF	Phillips-Perron
Panel A. Full Sample Period		
Total	− 1.574	− 0.908
ΔTotal	− 4.701***	− 18.178***
AA	− 0.847	− 0.918
ΔAA	− 7.600***	− 25.686***
Whites	− 1.604	− 1.000
ΔWhites	− 4.986***	− 19.515***
Latin	− 1.104	− 1.125
ΔLatin	− 7.081	− 25.284***
Panel B. Post 911 Subsample Period		
Total	− 1.396	− 1.096
ΔTotal	− 2.872***	− 8.914***
AA	− 1.578	− 1.967
ΔAA	− 3.798***	− 13.088***
Whites	− 1.322	− 1.231
ΔWhites	− 3.550***	− 9.485***
Latin	− 0.957	− 1.280
ΔLatin	− 3.879***	− 12.414***
Panel C. Financial Crisis—Inception to Peak		
Total	− 1.273	− 1.499
ΔTotal	− 1.218	− 3.356**
AA	− 1.246	− 1.293
ΔAA	− 2.123**	− 6.702***
Whites	− 1.404	− 1.547
ΔWhites	− 1.133	− 3.774***
Latin	− 1.435	− 1.597
ΔLatin	− 1.591	− 6.546***
Panel D. Post Financial Recession		
Total	− 3.451**	− 2.969**
ΔTotal	− 6.148***	− 14.607***
AA	− 1.058	− 1.077
ΔAA	− 5.719***	− 17.790***
Whites	− 3.560***	− 3.125**
ΔWhites	− 5.919***	− 14.992***
Latin	− 2.802	− 2.883
ΔLatin	− 5.574***	− 13.985

**Table 4:** Johansen cointegration tests for unemployment rates in the U.S

Max Rank	Parameters	LL	Trace	5% Critical
Panel A. Full sample period				
0	30	− 113.10	27.685*	29.68
1	35	− 101.92	5.32	15.41
2	38	− 100.16	1.81	3.76
Panel B. Pre 9/11 Subsample Period				
0	30	− 48.565	27.437*	29.68
1	35	− 39.951	10.208	15.41
2	38	− 34.847	0.442	3.76
Panel C. Post 9/11 Subsample Period				
0	30	4.507	44.521	29.68
1	35	18.468	16.598	15.41
2	38	24.983	3.567*	3.76
3	39	26.767		
Panel D. Pre 2008 Crisis Subsample Period				
0	30	− 6.583	28.523*	29.68
1	35	2.209	10.944	15.41
2	38	7054	1.254	3.76
3	39	7.681		
Panel E. Post 2008 Crisis Subsample Period				
0	30	− 6.583	28.523*	29.68
1	35	2.209	10.944	15.41
2	38	7054	1.254	3.76
3	39	7.681		

**Fig. 2:**
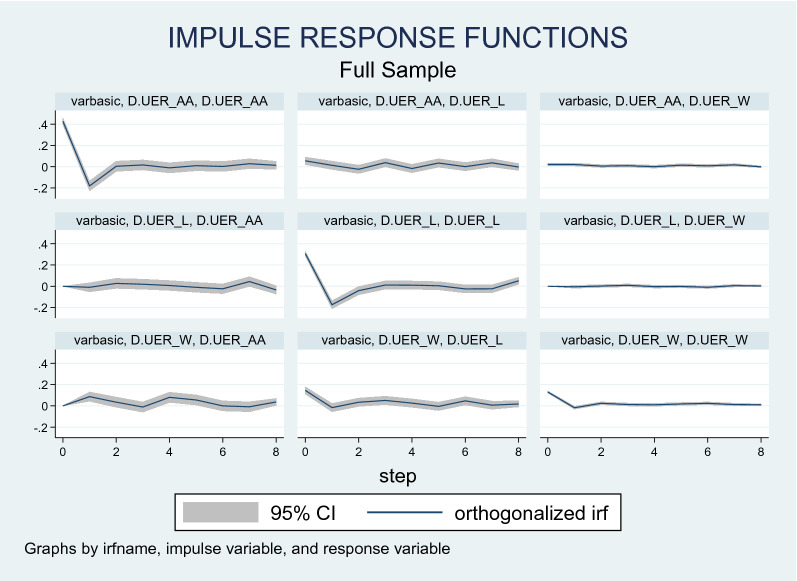
Impulse response functions

We also use the impulse response function to quantify the responsiveness of employment variables to structural changes in the system. Figure 2 depicts the response of different racial groups (white, African American, and Latin) to a shock in unemployment and per capita income. Figure 2 suggests that a one-standard deviation shock to the White unemployment sector causes a positive effect in the African American unemployment for 4 subsequent months. The same effect occurs for shocks emanating from the Latino sector as well, albeit not to the same magnitude.

To test for short-run causality, Table 5 reports the Vector Autoregressive (VAR) results^4^ which suggest that there is short-run causality running from White unemployment to African unemployment. This finding is corroborated by the Granger Causality test results in Table 6. In a nutshell, White unemployment Granger causes unemployment in the Latin and African American communities and Latino unemployment ganger cause African American Unemployment. We can also see causality running from the African American sector to the white sector.

**Table 5:** Vector Autoregressive Regression for Unemployment Rates by Race

	Full Sample Period				Pre-2008 Sample Period		
	ΔUER_AA_t_	ΔUER_W_t_	ΔUER_L_t_		ΔUER_AA_t_	ΔUER_W_t_	ΔUER_L_t_
ΔUER_AA_t-1_	.− 453*** (.053)	.056*** (.016)	.079 (.042)		.− 439*** (.067)	.055*** (.019)	.126** (.057)
ΔUER_AA_t-2_	− .253*** (.059)	.037** (.018)	− .007 (.047)		− .239*** (.074)	.044** (.021)	.000 (.064)
ΔUER_AA_t-3_	− .123** (.060)	.024 (.019)	.025 (.048)		− .161** (.076)	.030 (.019)	.001 (.065)
ΔUER_AA_t-4_	− .116 (.060)	.003 (.019)	− .063 (.048)		− .170** (.076)	.000 (.021)	− .062 (.065)
ΔUER_AA_t-5_	− .105 (.060)	.025 (.019)	.009 (.048)		− .113 (.076)	.011 (.021)	− .028 (.065)
ΔUER_AA_t-6_	− .100	.022	− .008		− .141*	.011	− .039
	(.058)	(.018)	(.046)		(.073)	(.021)	(.063)
ΔUER_AA_t-7_	− .032 (.052)	.037 (.016)	.063 (.042)		− .035 (.066)	.031 (.019)	.097* (.057)
ΔUER_W_t-1_	.670*** (.188)	− .116** (.058)	.503*** (.151)		.620** (.262)	− .175** (.073)	.381* (.225)
ΔUER_W_t-2_	.583***	.133**	.697***		.312	.121	.517**
	(.196)	(.061)	(.158)		(.270)	(.076)	(.232)
ΔUER_W_t-3_	.021 (.200)	.072 (.062)	.787*** (.161)		.307 (.272)	.073 (.076)	.889*** (.234)
ΔUER_W_t-4_	.417** (.205)	.059 (.063)	.614*** (.165)		.544* (.280)	− .019 (.078)	.664*** (.240)
ΔUER_W_t-5_	.594*** (.206)	.097 (.064)	.232 (.166)		.279 (.281)	.035 (.079)	.115 (.241)
ΔUER_W_t-6_	.197 (.185)	.149** (.063)	.424*** (.163)		.101 (.274)	.217*** (.077)	.820*** (.235)
ΔUER_W_t-7_	− .367 (.194)	.036 (.060)	.251 (.156)		− .346 (.272)	.077 (.076)	.290 (.234)
ΔUER_L_t-1_	− .031 (.071)	− .016 (.022)	− 564*** (.057)		− .036 (.084)	− .022 (.024)	− .602*** (.072)
ΔUER_L_t-2_	.068 (.080)	− .001 (.025)	− .442*** (.065)		.131 (.094)	− .023 (.026)	− .461*** (.081)
ΔUER_L_t-3_	.134 (.085)	.026 (.026)	− .287*** (.069)		.187 (.099)	.018 (.029)	− .318*** (.085)
ΔUER_L_t-4_	.132 (.086)	.002 (.027)	− .176** (.070)		.209** (.101)	.008 (.028)	− .150** (.086)
ΔUER_L_t-5_	.090	− .010	− .101		.206**	.001	− .087
	(.083)	(.026)	(.067)		(.096)	(.027)	(.083)
ΔUER_L_t-6_	.007 (.078)	− .035 (.024)	− .132 (.063)		.006 (.091)	− .029 (.025)	− .168** (.078)
ΔUER_L_t-7_	.131 (.069)	− .003 (.021)	− .166*** (.055)		.135 (.080)	− .008 (.022)	− .176*** (.069)
Constant	− .021 (.023)	.000 (.007)	− .019 (.018)		− .018 (.029)	.002 (.008)	− .027 (.025)
N = 36; Standard error in parentheses; **5% sig level; ***1% sig level							
JBera Test	.778	.010	.032		0.576	.327	.381
Lagrange Multiplier Test ($${H}_{0}: No autocorrelation at lag order$$)							
Lag 1 ${\chi }^{2}$	4.64 (.864)				Lag 1 ${\chi }^{2}$	1.74 (.995)	
Lag 2 ${\chi }^{2}$	9.77 (.369)				Lag 2 ${\chi }^{2}$	10.11 (.341)	
Lag 3 ${\chi }^{2}$	2.68 (.978)				Lag 3 ${\chi }^{2}$	1.97 (.991)	
Lag 4 ${\chi }^{2}$	3.69 (.931)				Lag 4 ${\chi }^{2}$	2.20 (.988)

**Table 6:** Granger Causality for Unemployment Rates by Race in the US

Equation	Excluded	${\chi }^{2}$	df	Prob > ${\chi }^{2}$
Panel A. Granger Causality Tests—Full Sample				
ΔUER_AA	ΔUER_W	31.17	7	0.000
	ΔUER_L	9.110	7	0.245
	ALL	70.77	14	0.000
ΔUER_W	ΔUER_AA	17.54	7	0.014
	ΔUER_L	6.10	7	0.528
	ALL	22.89	14	0.062
ΔUER_L	ΔUER_AA	11.19	7	0.131
	ΔUER_W	55.74	7	0.000
	ALL	88.42	14	0.000
Panel B. Granger Causality Test—Pre 2008 Sample				
Equation	Excluded	${\chi }^{2}$	df	Prob > ${\chi }^{2}$
ΔUER_AA	ΔUER_W	12.12	7	0.097
	ΔUER_L	13.79	7	0.055
	ALL	39.52	14	0.000
ΔUER_W	ΔUER_AA	12.35	7	0.014
	ΔUER_L	5.823	7	0.528
	ALL	17.69	14	0.062
ΔUER_L	ΔUER_AA	11.19	7	0.141
	ΔUER_W	55.74	7	0.000
	ALL	88.42	14	0.000
Panel C. Granger Causality Test—Post 2008 Sample				
Equation	Excluded	${\chi }^{2}$	df	Prob > ${\chi }^{2}$
ΔUER_AA	ΔUER_W	9.22	2	0.010
	ΔUER_L	2.31	2	0.314
	ALL	9.27	4	0.055
ΔUER_W	ΔUER_AA	1.78	2	0.412
	ΔUER_L	0.55	2	0.758
	ALL	2.13	4	0.713
ΔUER_L	ΔUER_AA	0.916	2	0.632
	ΔUER_W	5.657	2	0.059
	ALL	6.749	4	0.050

### Level shock analysis—the case of Illinois

**Fig. 3:**
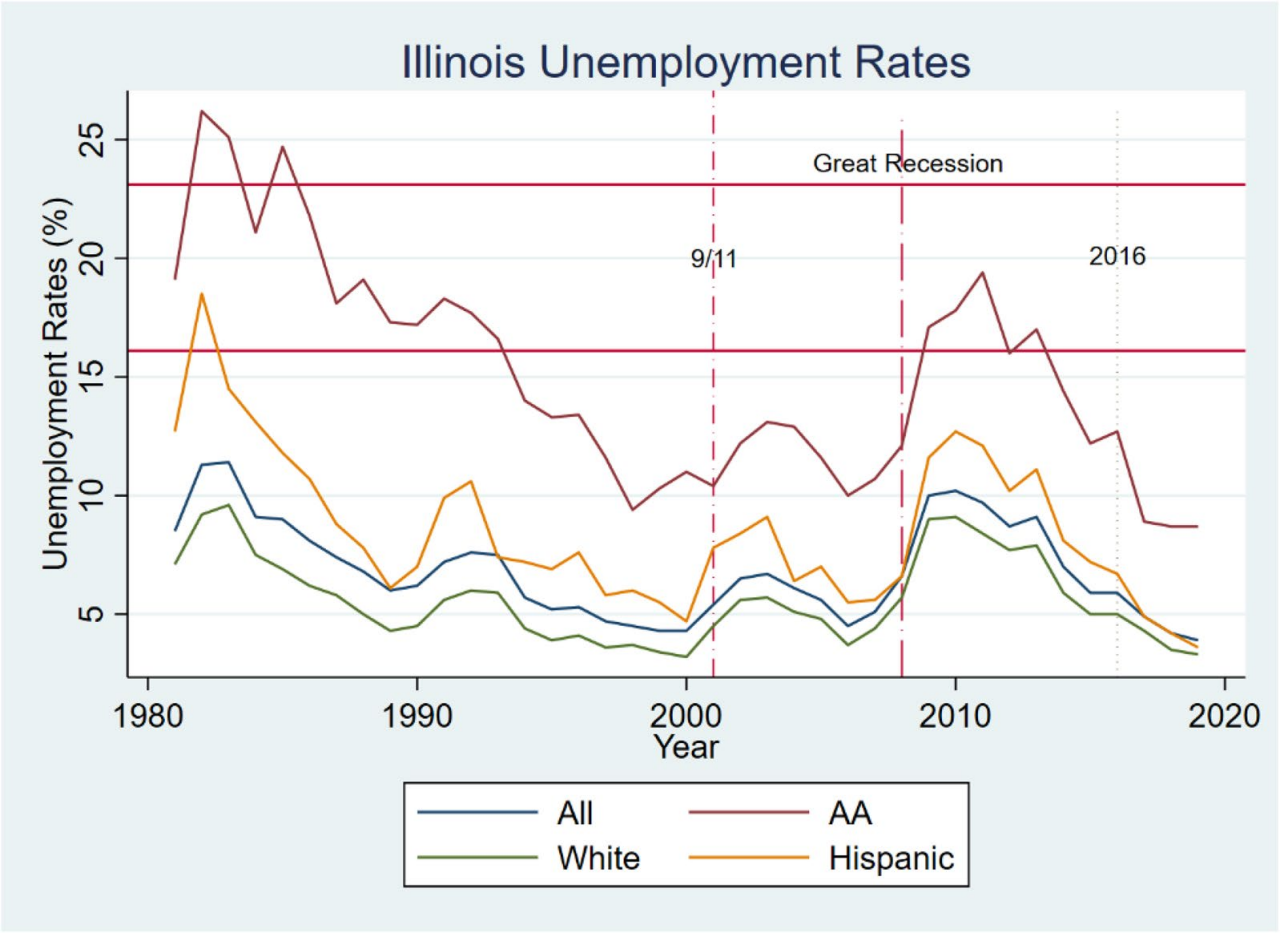
Illinois unemployment rates by race

Moving on to our analysis for the State of Illinois, Table 7 provides a summary of descriptive statistics for the unemployment rates for the State of Illinois, and Fig. 3 plots the time series fluctuations of Illinois unemployment rates defined by race. Unambiguously, the unemployment rates in Illinois are higher than the national averages for all racial groups. The mean unemployment rate for the African American sector is 15.2%, compared to 5.60% for the White sector, representing a multiple of 2.71 of African American to white unemployment. African Americans performed far worse on same-sector comparison of national to Illinois. The mean unemployment rate for African Americans in Illinois is higher by a multiple of 1.43, compared to 1.10 for the white sector. The Hispanic sector has a mean unemployment rate of 8.50%. Similarly, it was much higher than the national unemployment rate by a multiple of 1.07. The standard deviation of the unemployment rates for the full sample in Illinois is higher than they are for the national unemployment rates. The standard deviation of the unemployment rates for the African American sector is 4.68%, compared to only 1.82% for the White sector. Again, this was more than twice as volatile as the white sector, and higher than the Hispanic laborers who experienced a standard deviation of 3.19%. Clearly, the white sector’s market is more stable than the other two markets.

**Table 7:** Descriptive statistics on the unemployment rates for the State of Illinois

	African American	White	Latin	Total
Panel A. State of Illinois full sample period—Jan/1/1989 to 2/1/2020				
N	39	39	39	39
Mean	15.2	5.60	8.50	6.82
Median	14.0	5.10	7.60	6.50
S.D	4.68	1.82	3.19	2.04
Max.	26.2	9.6	18.5	11.4
Min.	8.7	3.2	3.60	3.9
Panel B. 9/11 subsample period				
Pre-9/11 period—1989 to 2001				
N	13	13	13	13
Mean	13.88	4.39	7.12	5.68
Median	13.40	4.30	7.00	5.40
S.D	3.21	0.92	1.66	1.17
Max.	18.30	6.00	10.60	7.60
Min.	9.40	3.20	4.70	4.30
Post-9/11 sample—2001 to 2008				
N	8	8	8	8
Mean	11.63	4.94	7.05	5.81
Median	11.85	4.95	6.80	5.85
S.D	1.16	0.72	1.29	.79
Max.	13.10	5.70	9.10	6.70
Min.	10.00	7.60	5.50	4.5
Panel C. 2008 global financial crisis subsample period				
Crisis period—2009 to 2010				
N	11	11	11	11
Mean	13.90	6.28	8.40	7.22
Median	14.40	5.90	8.10	7.00
S.D	3.92	2.20	3.32	2.40
Max.	19.40	9.10	12.70	10.20
Min.	8.70	3.30	3.60	3.90

Table 8 reports the mean differential for unemployment rates across various racial groups in Illinois before and after each recession. The figures show that Illinois benefitted well post 9/11 and 2008 crises. After the 9/11 crisis, African Americans saw a drop of 2.25% in their mean unemployment rates. This is much higher than the white sector that experienced 0.98% decrease in mean unemployment rates. However, during the 2008 crisis, African Americans experienced a 2.27% increase in the mean unemployment rates, compared to 1.34% by the white sector.

**Table 8:** Mean differential analysis for the unemployment rates for the State of Illinois

	Pre-911 UER	Post-911 UER	Mean Differential	t-statistic (p-value)
Panel A. Pre-911- Illinois Post 911 Means Differential analysis				
A.1. African Americans				
Mean	13.88	11.63	− 2.25	− 2.29 (.02)
S.D	3.21	1.16		
N	13	8		
A.2. White Americans				
Mean	5.60	4.62	− 0.98	− 1.84 (0.04)
S.D	1.82	.48		
N	13	8		

	Max 2008 UER	Post-2008 UER	Mean Differential	t-statistic (p-value)
Panel B. Pre-2008- Post 2008 Means Differential analysis				
B.1 African Americans				
Mean	11.63	13.9	2.27	1.81 (0.04)
S.D	1.16	3.92		
N	8	11		
B.2. White Americans				
Mean	4.94	6.28	1.34	1.88 (0.04)
S.D	0.72	2.20		
N	8	11		

### Labor market dynamics—the case of Illinois

To test the hypothesis that the demand for labor starts in the white sector in the state of Illinois, as it is believed to exist nationally, we look for cointegration among the African American and white unemployment rates. In unreported results, the results of the Augmented Dickey-Fuller and Phillips-Perron unit root tests confirm that all unemployment rate and GDP series are non-stationary in their levels and stationary in their first-differences. Also, the results of the Johansen Cointegration test suggest a maximum rank of order 2. Two series are said to be cointegrated if they are non-stationary in their levels, but stationary in their first differences. Using this outcome, we run a vector error-correction model in Table 9. The error-correction coefficients are statistically significant and negative at the 5% level. This suggest that white, Hispanic, and real GDP Granger-cause African American unemployment in the long run. Long run equilibrium is controlled by two error correction functions. Results show that 112% of the deviation from the long-run equilibrium in the African American unemployment is restored in the first in the first month after experiencing a shock by unemployment in the Latin community and GDP. This is followed by another correction of 320% of the disequilibrium from long-run equilibrium by the other error-correction equation.

**Table 9:** Illinois vector error-correction model

	African American Unemployment ΔUER_AA_t_	Whites Unemployment ΔUER_W_t_	Latin Unemployment ΔUER_L_t_	GDP ΔGDP_L_t_
ce1_t-1_	− 1.12** (.521)	.029 (.270)	− .675 (.433)	− 3989 (2941)
ce2_t-1_	3.20** (1.41)	− .000 (.000)	− .000** (.000)	− .093 (.079)
ΔUER_AA_t-1_	.117 (.337)	.027 (.175)	.471 (.280)	228 (1903)
ΔUER_AA_t-2_	− .04 (.252)	.126 (.131)	.398 (.209)	− 363 (1420)
ΔUER_W_t-1_	− 2.34 (1.255)	− .320 (.650)	− 1.60 (1.042)	− 2913 (7083)
ΔUER_W_t-2_	− .453 (1.11)	− .066 (.577)	− .953 (.924)	− 5102 (6280)
ΔUER_L_t-1_	1.085** (.430)	.371 (.223)	.545 (.357)	649 (2428)
ΔUER_L_t-2_	.173 (.359)	0.050 (.186)	.160 (.298)	649 (2428)
ΔGDP_L_t-1_	− .000 (.000)	− .000 (.000)	− .000 (.000)	.407 (.286)
ΔGDP_L_t-2_	− .000 .000	.000 (0.000)	.000 (.000)	− .068 (.297)
Constant	− .954 (.959)	.502 (.497)	.453 (.796)	.002 (5413)
Normality Test Jarque–Bera ${X}^{2}$ (p-value)	.719 (.697)	.960 (.619)	1.280 (.527)	1.617 (.446)
Autocorrelation ${X}^{2}$ (p-value)	Lag(1) 9.3375 (.899) Lag(2) 13.576 (.630)			

N = 36; Standard error in parentheses; **5% sig level; ***1% sig level

### Income differentials results

We now turn our attention to examine the unemployment and income differentials in the City of Chicago. Tables 10 and 11 provide descriptive statistics for households in 77 community areas in the city of Chicago, and for the 24 Community areas that makeup the city’s South and Southeast sides, respectively. The median income household across all 77 community areas is $53,392 compared to only $37,477 in the South and Southeast sides of Chicago. The disparity in income is exacerbated when comparing the maximum median income levels. The maximum median income for the entire city in 2019 was $111,962, compared to only $62,824 in the south/Southeast sides of the city. At the surface, households in the Southeast/South sides of the city earn 56% of the typical household across the city. Note also that the Southeast/South side of the city report the lowest median income ($15,030) in the city. As a further evidence, the average housing values (which are proxy of wealth) equal $254,850 in the city compared to $197,104 in the South/Southeast sides of Chicago. Again, note that the neighborhood area with the lowest housing values is also located in the South/Southeast sides of Chicago. Further, the Southeast/ South side of the city corresponds to the highest percentage of renters in the city. Over 50% of the Southeast/South side residences are renter occupied, compared with 47.2 across the city.

**Table 10:** Descriptive statistics for the 77 community areas in the City of Chicago

	N	Mean	Median	S.D	Max.	Min.
Household size	77	2.69	2.68	.59	4.3	1.53
Median income	77	$53,392	$50,178	$24,081	$111,962	$15,030
Unemployment rates_2019	77	8.5%	7%	5.5%	23.2%	1.9%
Employed in 2019	77	17,717	12,876	14,668	74,135	758
Population growth	77	-.03%	− .13%	.47%	2.04%	− .81%
House value	77	254,850	227,477	110,828	594,571	62,083
% Owner occupied	77	40.2%	36.4%	18.1%	79.8%	12.4%
% Renter occupied	77	47.2%	50.6%	15.9%	74.6%	13.8%
% vacancy	77	12.6%	10.1%	5.9%	32.4%	6.3%
% < HS Dip	77	16.2%	13.6%	10.0%	47.3%	1.4%
%w/HS Dip	77	25.3%	26.0%	9.9%	46.7%	4.4%
% W/Some college	77	25.9%	25.8%	8.4%	45.1%	8.2%
% w/Grad	77	32.7%	26.2%	21.9%	84.9%	5.4%
% w/White collar jobs	77	55.8%	52.8%	15.1%	89.1%	29.7%
% w/ service jobs	77	24.1%	24.8%	7.2%	39.8%	7.6%
% w/blue collar jobs	77	20.1%	19.6%	10.5%	45.5%	3.3%

**Table 11:** Descriptive Statistics for the 24 Community Areas in South and Southeast Areas of the City of Chicago

	N	Mean	Median	S.D	Max.	Min.
Household size	24	2.5	2.5	.39	3.34	1.8
Median income	24	$37,477	$34,518	$12,245	$62,824	$15,030
Unemployment Rates_2019	24	12.6%	12.8%	4.7%	22.3%	4.4%
Employed in 2019	24	8159	8439	5215	20,223	758
Population growth	24	− .1%	− .14%	.36%	.73%	− .81%
House Value	24	197,104	174,356	79,882	343,120	62,083
% Owner occupied	24	34.0%	29.6%	17.3%	66.8%	12.4%
% Renter occupied	24	51.0%	54.1%	16.2%	74.6%	23.7%
% vacancy	24	15.1%	15.8%	5.2%	24.8%	8.1%
% < HS Dip	24	15.2%	13.5%	6.7%	32.3%	3%
%w/HS Dip	24	26.6%	27.1%	7.4%	37.1%	6.4%
% W/Some College	24	31.8%	33.9%	8.3%	45.1%	13.5%
% w/Grad	24	26.4%	24.4%	15.3%	76.7%	6.7%
% w/White Collar Jobs	24	53.9%	52.7%	10.9%	83%	38.3%
% w/ Service Jobs	24	28.2%	29.0%	6.3%	39.8%	11.1%
% w/Blue Collar Jobs	24	17.9%	17.0%	7.5%	35.8%	5.8%

Community Areas: Chatham, Avalon Park, South Chicago, Burnside, Calumet Heights, Roseland, Pullman, South Deering, East Side, West Pullman, Riverdale, Hegewisch, Armour Square, Douglas, Oakland, Fuller Park, Grand Boulevard, Kenwood, Washington Park, Hyde Park, Woodlawn, South Shore, Bridgeport, Greater Grand Crossing

When it comes to educational attainment (schooling), 15.1% of the households within the South/Southeast sides have less than a high school diploma. In comparison, 16.2% of households within the city has attained less than a high school diploma. Households obtaining a high school diploma and some college, the South/Southeast sides report 58.4%, compared to 51.2% of households across the city. However, when it comes to obtaining a college degree or higher, the Southeast/Side sides reports only 26.4% of households, compared to 32.7% of the entire city. The mean unemployment rate in the City of Chicago was 8.5% in 2019, with a standard deviation of 5.5%. The maximum unemployment rate in the city was 3.2%. Compared to the city, the South/Southeast sides of the city had an average unemployment rate of 12.6%, almost 50% higher.

#### Earnings function

Specification 1 of Table 12 is a stylized estimate of Eq. (4). Grad, the percentage of households with a college degree, is the proxy for level of schooling. The coefficient of this variable is positive and statistically significant at the 1% level. A one unit increase in the percentage of households with at least a college increases the median income by 146%. A College degree explains 50% of the variation in median income. Specification (2) adds the dummy variable for households in the South/Southeast sides of the city. The coefficient is negative and statistically significant at the 1% level. This supports the common belief of wage and earnings suppression of African Americans (Nkomo and Ariss, 2014; Raymond, 2018; Mouw, 2000; Lynch and Hyclak, 2001; Immergluck, 1998). Controlling for educational attainment, households in the south/southeast sides of the city will have their median income reduced by 32.8%.

**Table 12:** Earnings function analysis

Dependent Variable: Log of Median Income		
	(1)	(2)
Grad	1.46*** (.144)	1.33*** (.127)
Southside	–	− .328*** (.076)
Constant	10.31*** (.065)	10.46*** (.067)
R^2^	.50	.61
N	77	77
AIC	45	27.5
RMSE	.320	.284
Normality Chi-Square test P-values in parentheses	1.96 (.38)	0.33 (.85)

Heteroskedasticity-Robust Errors in parenthesis

#### Essential workers

Table 13 presents the results of our analysis of the likelihood of being an essential worker. Specification 1 is the baseline equation. A one-unit increase in the percentage of households with high school diploma or less, increase the percentage of workers in the service sector. This level of schooling explains approximately 70% of the variation in percentage of workers in the service sector. Holding schooling constant, if a head of household is from the South/Southeast side of Chicago, there is an additional 3.5% likelihood of working as an essential worker. Specification 3 brings household income into the equation. Its coefficient is negative and statistically significant at the 1% level. A one percent increase in median income reduces the percentage of households working in the services sector by 6.1%. Again, if the households are in the South/Southeast sides of the City, they face a marginally higher likelihood of working as an essential worker, while controlling for schooling and income.

**Table 13:** Essential workers in the city of Chicago

Dependent % of variable: percentage of workers in services				
	(1)	(2)	(3)	(4)
No college degree	.28*** (.016)	.26*** (.016)	.19*** (.028)	.20*** (.029)
LN of Median Income	–	–	− .061*** (.016)	− .048*** (.017)
Southside	–	.035*** (.010)	–	.019** (.010)
Constant	.10*** .013)	.053*** (.010)	.768*** (.190)	.616*** (.199)
Heteroskedasticity-Robust Errors in parenthesis				
R^2^	.70	.75	.78	.79
N	77	77	77	77
AIC	− 272	− 281	− 289	− 289
RMSE	.039	.036	.035	.034
Chi-Square (P-values) Normality Test	4.73 (.09)	2.21 (.33)	13.65 (.00)	.68 (.71)

## Conclusion

In the research reported in the present study, our central finding is that firms in the labor market appear to prefer white employees to African American and Hispanics. This finding is attested by several interesting findings that emerge from our employment and income differential analyses.

Our employment differential analysis reveals that there is racial employment disparity which is first evident from the persistent near two-fold level of the national unemployment rates in the African American labor market. Over the full sample period, the unemployment in the African American sector is nearly twice that of the white sector, and we find this condition to be even larger in the City of Chicago, particularly the Southeast and South sides of the City. A similar pattern is observed in the two subsample periods surrounding the 9/11 terrorist attack and the 2007–2008 recession. While these two episodes experienced exogenous shocks to the labor market and led to significant increases in the unemployment rates in all sectors, the increase in unemployment rate in the white sector paled to that of the African American sector.

The major takeaway from our analysis is that there is a long-run association between white unemployment and African American unemployment, in the sense that white unemployment Granger-causes African American unemployment. That is, white unemployment experiences “natural-rate” even within aggregate demand gaps when the macro economy is not experiencing cyclical downturn. In contrast, African American unemployment is largely cyclical in nature, in the sense that the African American labor market appears to serve as a secondary labor market to the white sector that fills in during expansionary times but suffers great losses during economic downturns. The state of Illinois exhibits the same phenomenon, but to a greater level.

Moving onto our income differential analysis, we show that the African Americans in the south part of Chicago are more likely to have lower median incomes and they tend to work in the service sector of the economy, compared to their counterparts in other parts of the City. Until the COVID-19 pandemic, the service sector did not carry the “essential worker” moniker it has come to be known as. In fact, it was the sector that was considered low-skilled and was paid less in earnings. That sector of the labor force is typically female and non-unionized—particularly women of color. They now find themselves on the front line of the health battlefield without adequate personal protection equipment. This is now a sector of the labor market that arguably deserves hazard pay. These findings corroborate the narrative in the mainstream media that African Americans and women of color are paid less than white workers for doing the same jobs. Simply stated, African Americans are not paid the marginal product of their labor.

Our findings have important policy implications. While it is uncertain to know for sure what will be the effect of this purely healthy-related exogenous shock to the economy, the effect of the COVID-19 is certain to be deep for the African Americans who suffer from higher unemployment rates and lower median incomes. There is a great opportunity for local, state, and national leadership to alleviate the burden that the African American Community carries. To alleviate this expected hardship, targeted public policy should be introduced so that we must allocate funding and resources to where they are most needed, and policy recommendations must be reflective of this reality. A uniform policy approach will not address the varied needs of groups and communities given that people will differentially experience the initial and longer-term consequences of the viral pandemic social distancing protocols.

Hence, we propose two targeted policy recommendations. First, we recommend stimulating private fixed capital formation in African American communities. More specifically, we recommend providing guaranteed heavily subsidized loans to those investing in African American communities. An increase in capital expenditures in largely African American communities will increase economic output, increase and stabilize employment (decrease unemployment), increase household income, and increase local tax revenues. For maximum effectiveness, target industries that have the greatest leakages from those communities.

Our second recommendation is to enforce fair wages to ensure equitable wages across the labor markets. There is an abundance of evidence suggesting that the marginal product of labor is not compensated equitably across various sectors of the labor market. Unfair, below-market, wages to African Americans leads to a reduction in income, expenditures and savings in the African American community, which in turn reduces expected free cash flows to potential investors in the community, making investments less attractive. This contributes to an increase in unemployment that further decreases to household income—a vicious cycle. Reduced wage also reduces that individual’s propensity to repay interest on capital. This makes home ownership less likely and access to liquidity less likely. During economic downturn, a lack of liquidity increases hardship for the individual and for the community.

## Data Availability

All data will be available upon request.

## Footnotes

1 It is important to analyze the impact of both recessions separately, because this gives us a more clear-cut explanation of how two crises with different reasons may have different employment impact on the same market. While both exogenous shocks to the economy have had deleterious effects on the unemployment rates in general, their duration, and obviously causes, are different. On one hand, the September 11, 2001 attack was political in nature and unexpected by the population at large. Its aim was to place fear in the hearts of the American people, and the political reaction was swift as the Federal government moved to restructure the political system to ensure safety to the American people. Although the memories may be everlasting, the economy rebounded relatively quickly as the unemployment data suggest, given that the average unemployment rates took approximately 45 months to return to its pre-9/11 levels. This was in all account, a purely exogenous shock to an economy that was humming along. Hence, the uncertainty of coronavirus crisis is larger comparing to the September 11, 2001 recession that was caused by shorter analysis time. On the other hand, the 2007–2008 recession was an endogenous event that began in the real estate market and manifested into the global economy. This relatively painful shock took 92 months for the economy to return to its pre-shock level of unemployment. In comparison to the 2008 market downturn, COVID-19 crisis led consumers and firms all around the world to put off spending; they are in wait-and-see mode.

2 Note that it is also expected that the dynamics within the labor market may not be contemporaneous. If the shock is a negative, then unemployment will increase in the non-preferred sector of the labor market followed by an increase in the preferred sector. Because negative exogenous shocks are typically followed by fiscal and monetary policies, this will lead to an immediate reduction in the preferred sector of the labor market followed by a reduction in the non-preferred sector. Therefore, exogenous negative shocks and subsequent positive fiscal treatments affect both sectors in magnitude and speed of adjustments. Negative shocks begin with an increase in unemployment rates of the non-preferred leading to an increase in the unemployment rates of the preferred sector. Positive treatments affect the market in the opposite direction. This is known as feedback effect between the two sectors of the labor market.

3 The subsample for the post-911 sample suggests cointegration with a rank = 2. However, the sample size has only 9 observations. This is too small to perform any meaningful time series analysis.

4 We use the first-differenced data in this VAR model. We use a hybrid of 5 lag length tests (LR, FPE, and three Information criteria tests) to determine the optimal lag lengths, as they differ according to the sample size.

## References

[CR1] Aali-Bujari A, Venegas-Martinez F, Garcia-Santillan A (2019). Schooling levels and wage gains in Mexico. Econ. Sociol..

[CR2] Abdul Khalid M, Yang L (2021). Income inequality and ethnic cleavages in Malaysia: Evidence from distributional national accounts (1984–2014). J. Asian Econ..

[CR3] Becker GS (1962). Investments in human capital: a theoretical analysis. J. Polit. Econ..

[CR4] Bond TN, Lehmann JK (2018). Prejudice and racial matches in employment. Labour Econ..

[CR5] Boustan LP, Margo RA (2009). Race, segregation, and postal employment: new evidence on spatial mismatch. J. Urban Econ..

[CR6] Button P, Walker B (2020). Employment discrimination against Indigenous Peoples in the United States: Evidence from a field experiment. Labour Econ..

[CR7] Chantreuil F, Fourrey K, Lebon I, Rebiere T (2021). Magnitude and evolution of gender and race contributions to earnings inequality across US regions. Res. Econ..

[CR8] Chiswick BR (2003). Jacob Mincer, Experience and the Distribution of Earnings. Rev. Econ. Household.

[CR9] Contreras S, Ghosh A, Hasan I (2021). Income inequality and minority labor market dynamics: Medium term effects from the Great Recession. Econ. Lett..

[CR10] Couch KA, Fairlie R, Xu H (2020). Early evidence of the impacts of COVID-19 on minority unemployment. J. Public Econ..

[CR12] Hellerstein JK, Neumark D, Mclnerney M (2008). Spatial mismatch or racial mismatch?. J. Labour Econ..

[CR13] Hoynes H, Miller DL, Schaller J (2012). Who suffers during recessions. J. Econ. Persp..

[CR14] Ileanu BV, Tanasoiu OE (2008). Factors of the earning functions and their influence capital of an organization. J. Appl. Quant. Methods.

[CR15] Immergluck D (1998). Job proximity and the urban employment problem: do suitable nearby jobs improve neighborhood employment rates?: a reply. Urban Stud. J..

[CR16] Kim AT, Kim C, Tuttle SE, Zhang Y (2021). COVID-19 and the decline in Asian American employment. Res. Soc. Stratif. Mobility.

[CR17] Lynch GJ, Hyclak T (1984). Cyclical and noncyclical unemployment differences among demographic groups. Growth Chang..

[CR18] Macartney H, Nielsen E, Rodriguez V (2021). Unequal worker exposure to establishment deaths. Lab. Econ..

[CR19] Mandel H, Semyonov M (2021). The gender-race intersection and the ‘sheltering-effect’ of public-sector employment. Res. Soc. Stratif. Mob..

[CR20] Mincer J (1958). Investment in human capital and personal income distribution. J. Polit. Econ..

[CR21] Mouw T (2000). Job Relocation and the Racial Gap in Unemployment in Detroit and Chicago, 1980 to 1990. Am. Sociol. Rev..

[CR22] Nkomo SM, Ariss A (2014). The historical Origins of Ethnic (white) Privilege in US Organizations. J. Manag. Psychol..

[CR23] Raymond EL (2018). Race, uneven recovery and persistent negative equity in the southeastern United States. J. Urban Aff..

[CR24] Ren C (2022). Cohort, signaling, and early-career dynamics: The hidden significance of class in black-white earnings inequality. Soc. Sci. Res..

[CR25] Yu W, Sun S (2019). Race-ethnicity, class, and unemployment dynamics: do macroeconomic shifts alter existing disadvantages?. Mobility.

